# The ASIC3-M-CSF-M2 macrophage-positive feedback loop modulates fibroblast-to-myofibroblast differentiation in skin fibrosis pathogenesis

**DOI:** 10.1038/s41419-022-04981-9

**Published:** 2022-06-06

**Authors:** Jun-Jie Wu, Zi-Li Sun, Si-Yu Liu, Zhong-Hua Chen, Zheng-Dong Yuan, Ming-Li Zou, Ying-Ying Teng, Yue-Yue Li, Dan-Yang Guo, Feng-Lai Yuan

**Affiliations:** 1 Institute of Integrated Chinese and Western Medicine, The Hospital Affiliated to Jiangnan University, Wuxi, Jiangsu 214041 China; 2 The Hospital Affiliated to Jiangnan University, Wuxi, Jiangsu 214041 China; 3 Nanjing University of Chinese Medicine, Nanjing, Jiangsu 210000 China; 4 The Nantong University, Nantong, Jiangsu 226000 China

**Keywords:** Diseases, Cell biology

## Abstract

Inflammation is one of the main pathological features leading to skin fibrosis and a key factor leading to the progression of skin fibrosis. Acidosis caused by a decrease in extracellular pH is a sign of the inflammatory process. Acid-sensing ion channels (ASICs) are ligand-gated ion channels on the cell membrane that sense the drop in extracellular pH. The molecular mechanisms by which skin fibroblasts are regulated by acid-sensing ion channel 3 (ASIC3) remain unknown. This study investigated whether ASIC3 is related to inflammation and skin fibrosis and explored the underlying mechanisms. We demonstrate that macrophage colony-stimulating factor (M-CSF) is a direct target of ASIC3, and ASIC3 activation promotes M-CSF transcriptional regulation of macrophages for M2 polarization. The polarization of M2 macrophages transduced by the ASIC3-M-CSF signal promotes the differentiation of fibroblasts into myofibroblasts through transforming growth factor β1 (TGF-β1), thereby producing an ASIC3-M-CSF-TGF-β1 positive feedback loop. Targeting ASIC3 may be a new treatment strategy for skin fibrosis.

## Introduction

Skin fibrosis is a critical pathological factor that affects skin function and is a feature of fibrosing connective tissue diseases in the dermis, including hypertrophic scars and keloids [1]. Hypertrophic scars and keloids occur from cutaneous injury or skin stimulation that is deep enough to influence the dermal layer [2]. These disorders share standard structural features: a massive matrix deposition of collagen fiber and other extracellular matrix (ECM) proteins by activated dermal fibroblasts or myofibroblasts [3]. Although the exact mechanisms underlying skin fibrosis are still unclear, many factors are thought to influence the formation of skin fibrosis. Research has indicated that any condition promoting an inflammatory environment in the dermis can affect the degree of fibrosis [4].

Monocytes and macrophages are relatively abundant types of leukocytes that function in the early stage of inflammation [5]. As the wound environment changes, monocytes undergo differentiation into M1 or M2 phenotypes to exert different physiological functions. M1 macrophages exhibit pro-inflammatory effects [6], while M2 macrophages are present as inflammation begins to subside and mediate the repair process in tissue [7]. M2 macrophages produce transforming growth factor β1 (TGF-β1), which regulates the proliferation, differentiation, migration, and other physiological activities of dermal fibroblasts, and affect the repair of tissues [8]. In addition to the well-documented macrophage-mediated activation of skin fibroblasts, accumulating evidence indicates that fibroblasts, in turn, indirectly regulate macrophages via cytokines [9]. However, the molecular mechanisms by which the inflammatory environment of the dermis mediates the fibroblast-macrophage communication are not well understood.

A low pH tissue environment is a hallmark of a variety of inflammatory processes [10]. Acid-sensing ion channels (ASICs) [11] are the primary extracellular acid sensors in peripheral tissues and function as ligand-gated ion channels on the cell membrane that sense the drop in extracellular pH [12]. Six ASIC subunit proteins, encoded by four genes (*ASIC1* to *ASIC4*), have been identified: ASIC1a, ASIC1b, ASIC2a, ASIC2b, ASIC3, and ASIC4 [13–15]. These proteins are widely expressed in peripheral sensory neurons and non-neuronal cells involved in various physiological and pathological processes [16]. We previously showed that ASIC1a is an important extracellular acid sensor in bone cells that contributes to the apoptosis of chondrocytes and osteoclastogenesis [17, 18]. ASIC3 mRNA and protein are expressed in fibroblast-like synoviocytes, which may be important for the modulation of hyaluronan production within joint tissue [19]. ASIC3 acts as a pH sensor in fibroblast-like synoviocytes in joint tissues [20]. However, the molecular mechanism through which skin fibroblasts are regulated by ASICs has been unknown.

In this study, we demonstrated that ASIC3 is associated with skin fibrosis. ASIC3 showed a considerable difference in expression in fibrotic tissues compared with normal skin. In vivo and in vitro experiments revealed that exogenous promotion of the opening of the ASIC3 channel accelerated local M2 macrophage aggregation and skin fibrosis. In addition, we found that ASIC3 regulates the secretion of M-CSF by fibroblasts, and M2 macrophages transduced by the ASIC3-M-CSF signal promote the production of alpha-smooth muscle actin (α-SMA)-positive fibroblasts by TGF-β1, thereby producing the ASIC3-M-CSF-TGF-β1 positive feedback loop. These results have expanded our understanding of the mechanisms by which ASICs and macrophages regulate skin fibrosis.

## Materials and methods

### Patients and tissue samples

Ten keloid samples were obtained from patients with keloids; the patient group included five men and five women (mean age 25.4 years; range 18–38 years). Ten hypertrophic scar samples were obtained from patients with hypertrophic scar; the patient group included five men and five women (mean age 29.5 years; range 18–42 years). All the patients met the current diagnostic criteria for keloid/hypertrophic scar, defined as the presence of typical skin lesions confirmed by plastic surgeons or dermatologists. Ten normal skin samples were obtained adjacent to a normal scar during scar revision surgery. Tables S1 and S2 provide information on cases of hypertrophic scars and keloids. A complete list of the 20 cases is provided in Tables S1 and S2. All subjects signed an informed consent form approved by the local institutional review board (ethical approval was obtained from Affiliated Hospital of Jiangnan University, Wuxi, China).

### Cells and cell culture

Skin specimens were washed, cut into small pieces, and digested with 0.1% collagenase I for 4 h at 37 °C. Cells were then suspended in a culture medium (DMEM with 10% FBS, penicillin, and streptomycin) at 37 °C in 5% CO_2_. Cells at passages 2–5 were used in experiments.

THP-1 monocytes (a human monocytic cell line; Chinese Academy of Sciences Cell Bank) were maintained in RPMI 1640 medium (Gibco, USA) with 10% FBS (Gibco), 0.05 mM β-mercaptoethanol (Gibco), penicillin and streptomycin at 37 °C in 5% CO_2_. THP-1 monocytes were differentiated into M0 macrophages by culture with 100 nM phorbol 12-myristate 13-acetate (PMA, Sigma, USA) in RPMI medium for 48 h.

For co-culture experiments, human skin fibroblasts were plated at a density of 5 × 10^4^ cells/mL on a 12-well plate in the DMEM medium and 5 × 10^5^ cells/mL of M0 macrophages were seeded on the membrane of the Transwell insert (Corning, USA) in RPMI 1640 medium. After 24 or 48 h incubation, fibroblasts and macrophages were collected for assays.

### Animal models and treatment

All animal experimental procedures were approved by the Experimental Animal Committee of Jiangnan University. New Zealand white rabbits (2.2–2.5 kg each) were purchased from the Huishan Experimental Animal Center (license no.: SCXK 2015-0004; Wuxi, China). Each rabbit was anesthetized with 10 g/L sodium pentobarbital at 30 mg/kg, and six identical, 0.6-cm full-thickness circular wounds were created on the ventral surface of each ear using a 0.6-cm biopsy punch. The epidermis, dermis, and perichondrium in every wound bed were completely removed with a surgical blade. The wounds were exposed to air and cleaned by the removal of secretions for 2 days. The rabbits were divided into three groups using random number: control group, vehicle group, and GMQ group, GMQ (2 mM, 50 μl) or DMSO (50 μl) was injected into each wound from day 7 after wounding; the control group received no treatment. The wound tissues were collected on days 7 and 14 for assays, and tissues were collected on day 21 after scar formation.

### Lentivirus transduction and overexpression of ASIC3

The lentivirus for ASIC3 expression was constructed by GeneChem (Shanghai, China). Fibroblasts were seeded on 6-well plates (2 × 10^5^/well) and cultured for 16 h. The medium was changed to fresh medium containing 40 μl HitransG A/HitransG P (GeneChem) and 20 μl lentivirus suspension (ASIC3-expressing lentivirus or blank lentivirus as the control), and the fibroblasts were incubated at 37 °C. After 24 h incubation, the medium was replaced, and the cells were collected for follow-up experiments.

### Western blotting

Total protein was extracted using a Total Protein Extraction Kit (Sangon, China), and protein concentration was measured with a BCA Protein Assay Kit (Beyotime, China). Protein samples were separated by 10% SDS polyacrylamide gels and electrotransferred onto polyvinylidene fluoride (PVDF) membranes (Millipore, USA). PVDF membranes were blocked and then incubated with polyclonal antibodies against ASIC3 (Alomone Labs, Israel, ASC-018), α-SMA (CST, USA, D4K9N), Collagen I (Abcam, USA, ab138492), CD206 (Novus Biologicals, USA, MAB2534), PI3K (Abcam, USA, ab191606), p-PI3K (Abcam, USA, ab182651), AKT (Abcam, USA, ab179463), p-AKT (Abcam, USA, ab38449), or GAPDH (Abcam, USA, ab8245) overnight at 4 °C and then incubated with horseradish peroxidase-conjugated secondary antibody (Abcam, USA, 7074) at room temperature for 1 h. GAPDH served as an internal control for protein normalization.

### Reverse transcription and quantitative real-time PCR (qRT-PCR)

Total RNA was isolated from cells and tissue using TRIzol (Thermo, USA) and then reverse transcribed at 37 °C with the PrimeScriptTM RT reagent Kit (Takara, Japan). Gene expression was measured by real-time PCR with TB Green® Premix Ex TaqTM II (Takara) on a 7500 RT-PCR instrument (Applied Biosystems, Foster City, CA, USA). GAPDH was used as endogenous control and the 2^−ΔΔCt^ method was used to determine relative gene expression. A complete list of primer sequences is provided in Table S3.

### Histological analysis and immunostaining

Biopsies of the wound or scar tissue were excised, fixed, embedded in paraffin, sectioned into 4-μm slices, and stained with an H&E staining kit (Beyotime) and Masson’s trichrome (Solarbio, China). The scar elevation index (SEI) was used to quantify scar formation and calculated as the ratio of the total scar area to the area of the normal underlying dermis. The SEI measurements were performed using the HE-stained tissue sections at a magnification of ×100. An SEI of 1 indicated normal healing without hypertrophic dermis, while an SEI of >1 indicated HS. The SEI was measured using Image-Pro-Plus version 6.0.

Tissue sections (4-μm-thick) were used for immunostaining. After dewaxing and rehydration, the tissue sections were treated with preheated antigen retrieval buffer (sodium citrate buffer) at 95 °C for 10 min, followed by washing with PBS and blocking with goat serum at 37 °C for 30 min. Tissue sections were blocked and then incubated with antibodies against ASIC3 (Alomone Labs, Israel, ASC-018) overnight at 4 °C followed by secondary antibody (Abcam, USA, 7074) for 1 h at room temperature. According to the protocol in the IHC reagents kit (Boter, China, AR1027-1). Images were obtained using a fluorescent inverted microscope (Olympus, Japan).

### Immunofluorescence

Cells were seeded at 40% confluence on round glass slides in 24-well plates. Cells were then fixed with 4% paraformaldehyde for 15 min at room temperature, followed by permeabilization with 0.1% Triton X-100 (Beyotime) in PBS. After blocking in goat serum at 37 °C for 30 min, the samples were incubated with antibodies against CD68 (Novus Biologicals, USA, NBP2-32831), CD206 (NovusBiologicals, USA, MAB2534), α-SMA (Novus Biologicals, USA, NBP2-33006), or Collagen I (Novus Biologicals, USA, NB600-408) in Diluent at 4 °C overnight. The samples were then incubated with secondary antibody (CST, USA, 8889/4412) at 37 °C for 1 h, followed by washing with PBS and staining with DAPI for nuclear visualization. Cell staining was examined under an inverted fluorescent microscope. The quantification of the mean fluorescence intensity (MFI) was performed using Image-Pro-Plus version 6.0.

### Flow cytometry

After 48 h treatment, macrophage phenotypes were analyzed by flow cytometry. M2 macrophages were identified using antibodies specific to CD206 (Biolegend, USA; 321104). For phenotypic analysis, macrophages were suspended in PBS at 1 × 10^6^/ml. Cell suspensions were incubated for 20 min with goat serum and then incubated with the diluted antibodies for 30 min on ice. Macrophages were finally suspended in cell staining buffer for flow cytometry. Data were acquired using a BD FACSCalibur (BD Biosciences, USA) with Flowjo software (Tree Star, USA).

### Cytotoxicity assay

The CCK8 method was used to measure the cytotoxicity of GMQ at different concentrations. Fibroblasts were grown in a 96-well plate (2 × 10^3^ cells/well) for 24 h. The culture supernatant was replaced with a maintenance medium containing GMQ at different concentrations. After 6, 12, or 24 h incubation, the culture supernatant was removed, and 190 μl DMEM and 10 μl CCK8 were added to each well. The samples were incubated at 37 °C for 2 h, and a microplate reader was used to determine the fluorescence intensity at 450 nm.

### Wound-healing assay

Fibroblasts were cultured in a 6-well plate. After 24 h treatment, a 10 µl tip was used for scratching the cell monolayer and the media was replaced with DMEM with no serum. The scratched area was photographed after 48 h, and the wound width was calculated using Image-Pro-Plus v6.0.

### Gel contraction assay

Fibroblasts were mixed with collagen I solution at a concentration of 1 × 10^5^/mL and then 15 μl of 0.5 N NaOH was added per ml. The cell mixture was immediately plated into 24-well plates (500 μl per well), and the plates were incubated in a cell culture incubator for 30 min. The polymerized gel was detached with a pipette tip, and 500 μl of complete growth medium was added to each well. For neutralization experiments, neutralizing antibodies were added to the co-culture system for 24 h (R&D Systems); the neutralizing dose (ND50) was 1 μg/ml. After 48 h, photos were taken, gel shrinkage activity was measured with ImageJ software, and the gel surface area was calculated from the ratio of the bottom area of the hole.

### Enzyme-linked immunosorbent assay (ELISA)

The expression levels of cytokines in cell culture supernatants were detected using the quantikine human ELISA kit (R&D Systems) as previously described [21].

### Cytokine antibody array

The expression of 80 cytokines was examined by a cytokine antibody array. Briefly, after 24 h, the cell supernatant of each treatment group was collected and the cytokine array was performed to evaluate the expression of 80 cytokines. Evaluations using the human inflammation antibody array (AAH-CYT-G5) were carried out by Shanghai Wayen Biotechnology Corporation (China) following standard protocols, as previously described [22]. A complete list of the 80 cytokine antibody array distributions is provided in Table S4.

### RNA sequencing

Total RNA was isolated as described above for RNA-seq analysis. cDNA library construction and RNA-seq were performed by GeneChem (Shanghai, China). RNA-seq was performed using an Illumina HiSeq4000 system. Gene expression was assessed by fragments per kilobase of transcript per million fragments mapped. Differentially expressed genes were screened using the DESeq2 algorithm. Genes with an adjusted *P* < 0.05 and fold change >2 were regarded as differentially expressed genes and subjected to subsequent enrichment analysis.

### Statistical analysis

Each independent experiment was performed at least three times. Data are shown as mean ± standard deviation (SD). Differences between the two groups were compared by Student’s *t*-test. Multiple groups were compared by one-way ANOVA. *P* < 0.05 was considered to be statistically significant.

## Results

### High ASIC3 expression correlates with skin fibrosis in hypertrophic scar and keloid

**Fig. 1:**
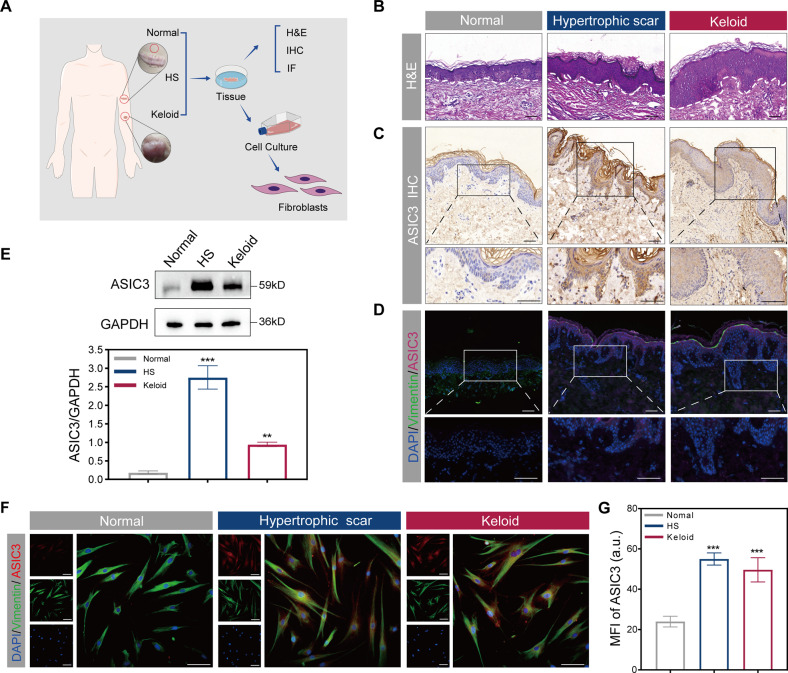
ASIC3 is highly expressed in human hypertrophic scar/keloid tissues and human primary cells. **A** Schematic diagram of sample source and experimental analysis. **B** Representative H&E staining images of human normal skin, hypertrophic scar (HS), and keloid. Scale bars, 100 μm. **C** Immunohistochemistry of ASIC3 expression in normal skin, hypertrophic scar and keloid. Scale bars, 100 μm and 50 μm. **D** Immunofluorescence analysis of ASIC3 (red) and vimentin (green) expression in human normal skin, hypertrophic scar, and keloid. Scale bars, 100 μm and 50 μm. **E** Western blot analysis of ASIC3 expression in normal skin, hypertrophic scar, and keloid. GAPDH served as loading control (*n* = 3). **F** Immunofluorescence analysis of ASIC3 (red) and vimentin (green) expressions in fibroblasts derived from normal skin, hypertrophic scar, and keloid. Scale bars, 50 μm. **G** MFI quantification of ASIC3 in (**F**) (*n* = 3). Data are expressed as the means ± SD. **P < 0.01 and ***P ≤ 0.001 compared with normal skin. HS, hypertrophic scar; ASIC3, acid-sensing ion channel 3; DAPI, 4′,6-diamidino-2-phenylindole; IHC, immunohistochemistry; IF, immunofluorescence; GAPDH, glyceraldehyde-3-phosphate dehydrogenase.

ASICs are essential acid sensors in fibroblast-like synoviocytes that are critical to the inflammatory process [19, 23]. We assessed the differential expression of ASICs in fibroblasts of fibrotic skin diseases, namely hypertrophic scars and keloids (Fig. 1A). We found that the expression of ASIC3 mRNA was upregulated in cutaneous fibrotic connective tissue disease (Fig. S1A). In contrast, the expression of other ASIC family members associated with skin fibrosis was not significantly different. As shown in Fig. 1B, both hypertrophic scar and keloid show a thicker epidermis than normal skin as determined by hematoxylin and eosin (H&E) staining. ASIC3 expression was increased in disease-derived dermal fibrosing tissue compared with normal skin as detected by immunofluorescence and immunohistochemistry (Fig. 1C, D). We examined ASIC3 expression in hypertrophic scar and keloid disease tissues by western blot and found that ASIC3 protein expression was upregulated in fibrotic tissues compared with normal skin (Fig. 1E). ASIC3 mRNA expression was significantly increased in hypertrophic keloids and keloid-derived fibroblasts compared with expression in normal fibroblasts (Fig. S1B), and increased ASIC3 protein expression was confirmed by immunofluorescence staining analysis (Fig. 1F, G). We also found that ASIC3 expression was positively correlated with hypertrophic keloids and collagen expression in keloids (Fig. S1D–E). These results suggest ASIC3 may act as an acid sensor to sense the acidic environment of skin inflammation and the increase of ASIC3 may have a causal relationship with fibrotic skin.

### ASIC3 promotes scar formation by macrophage recruitment

The small molecule 2-guanidine-4-methylquinazoline (GMQ) is an agonist of ASIC3 that activates and modulates the ASIC3 channel [19]. To test whether activated ASIC3 by GMQ exacerbates the fibrogenesis process in hypertrophic scar, we next performed CCK8 cytotoxicity assays in fibroblast cells treated with different concentrations of GMQ and found that GMQ induced toxicity of cells (Figure S1D). Based on the results, we selected the concentration of 20 μM GMQ for subsequent experiments.

**Fig. 2:**
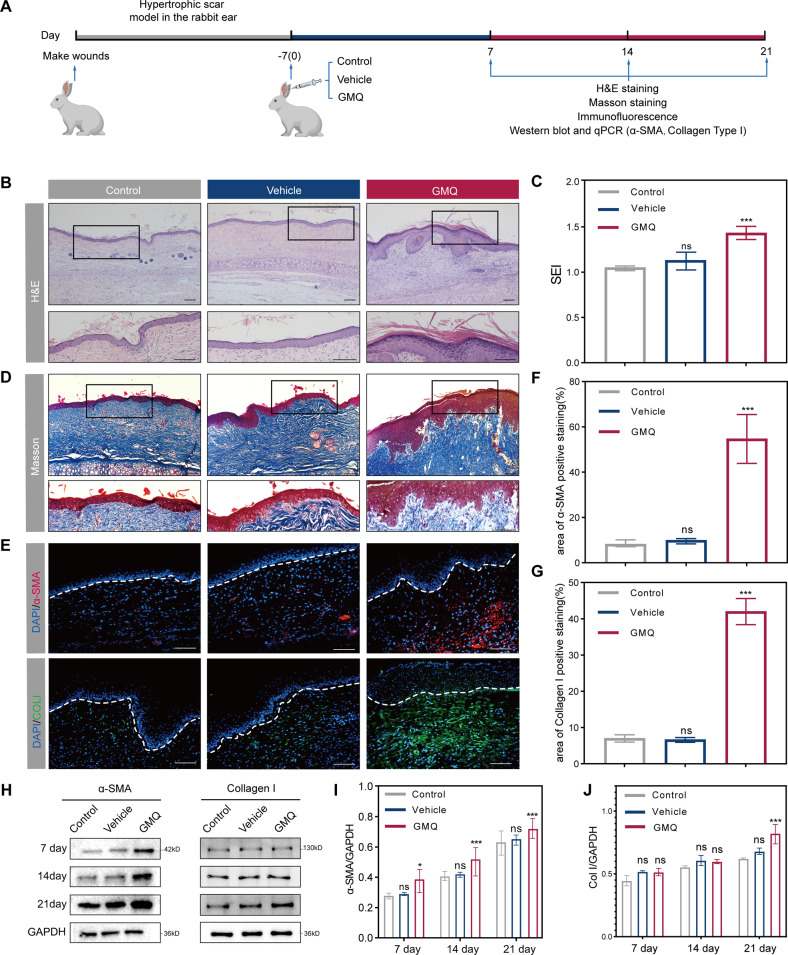
ASIC3 promotes scar formation in vivo. **A** Schematic diagram of the rabbit ear hypertrophic scar model. The model was subcutaneously administered either GMQ or DMSO. **B** Representative H&E staining images of rabbit ear tissue at day 21 after wounding. Scale bars, 100 μm. The black boxes in the upper panel are enlarged in the lower panel. Scale bars, 50 μm. **C** The scar elevation index (SEI) was significantly higher in the GMQ group than in the vehicle and control groups (*n* = 3). **D** Masson staining of rabbit ear tissue at 21 days after wounding. Scale bars, 100 μm. The black boxes in the upper panel were enlarged in the lower panel. Scale bars, 50 μm. **E** Immunofluorescence analysis of α-SMA (red) and collagen I (green) expressions in rabbit ear tissue 21 days after wounding (*n* = 3). The dotted line indicates the epidermis. Scale bars, 100 μm. **F**–**G** Quantification of the α-SMA- and collagen I–positive areas by immunofluorescence. **H**–**J** Western blot analysis of α-SMA and collagen I expression at 7 days, 14 days, and 21 days after wounding. GAPDH served as a loading control. Data are expressed as the means ± SD (*n* = 3). *P < 0.05, **P < 0.01, and ***P ≤ 0.001 compared with control. NS, not significant; α-SMA, α-smooth muscle actin; ColI, collagen I; GAPDH, glyceraldehyde-3-phosphate-dehydrogenase; GMQ, 2-guanidine-4-methylquinazoline.

We next examined the effects of stimulating ASIC3 after wounding in vivo. Rabbit ears were subcutaneously injected with GMQ for 7 days after the wound model was established (Fig. 2A). H&E-stained sections of rabbit ear tissue collected 21 days after wounding showed that numerous fibroblasts, collagen fibers, and inflammatory cells were increased in the healing wound in the GMQ treatment group; in addition, GMQ treatment increased the SEI (Fig. 2B, C). ECM deposition in the wound area was detected by Masson’s staining and increased on day 21 after GMQ injection (Fig. 2D). In addition, after GMQ treatment, the presence of α-SMA-positive cells and collagen type I (COL-I)-positive cells determined by immunofluorescence staining showed a significant increase in the healing wound (Fig. 2E–G). Collagen deposition is characterized by overexpression of COL-I, and increased contraction of myofibroblasts is marked by augmented α-SMA expression [24, 25]. As wound healing progressed, α-SMA and COL-I were assessed by western blot. We found that GMQ injection treatment markedly increased the levels of α-SMA and COL-I in the healing wound (Fig. 2H–J). These results indicate that ASIC3 may promote the formation of hypertrophic scar.

### ASIC3-mediated fibroblast-to-myofibroblast differentiation requires macrophages

The main pathological change underlying hypertrophic scar formation is the development of numerous myofibroblasts (activated fibroblasts) [26]. To explore whether ASIC3 activation is involved in fibroblast-to-myofibroblast differentiation, we used the agonist GMQ and pH 5.0 acidic solution to activate ASIC3 [27, 28] and examined α-SMA and COL-I expression to evaluate fibroblast-to-myofibroblast differentiation and ECM deposition indirectly. However, ASIC3 activation did not have significant effects on α-SMA and COL-I expression in fibroblasts (Figure S2). Consistent with these observations, acidic solution (pH 5.0), GMQ and ASIC3 overexpression (Figure S1G–I) did not increase α-SMA or COL-I mRNA (Figure S2A–C) or protein (Figure S2D–F) in fibroblasts. Wound-healing experiments showed that the migration of fibroblasts was significantly affected (Figure S2G, H), suggesting that ASIC3 was not directly involved in ECM protein deposition and cell migration.

**Fig. 3:**
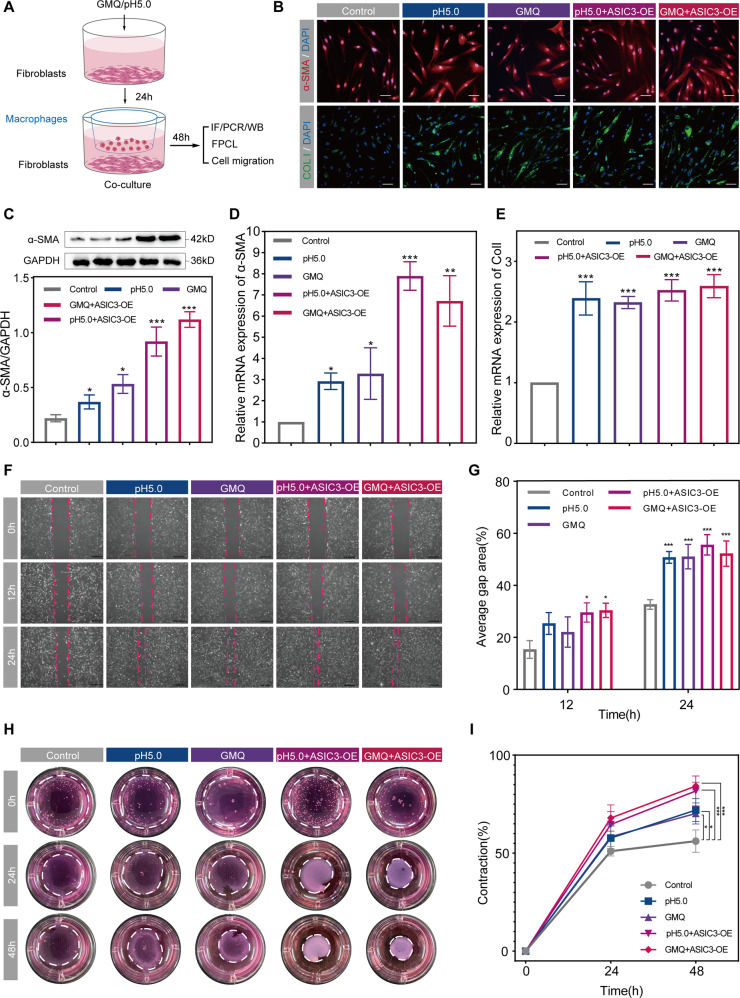
Activation of ASIC3 promotes fibroblast-to-myofibroblast differentiation through a process requiring macrophages. **A** Indirect co-culture cell model and experimental plan. **B** Immunofluorescence staining of α-SMA (red) and collagen I (green) in fibroblasts and control after 48 h of co-cultivation. Scale bars, 100 μm. **C** Western blot analysis of α-SMA in the indicated groups (*n* = 3). GAPDH served as a loading control. **D**, **E** α-SMA and collagen I mRNA levels after 48 h of co-cultivation (*n* = 3). **F**–**G** Wound-healing assays of fibroblast migration at 12 or 24 h after wounding (*n* = 3). Scale bars are 100 μm. **H**, **I** The contractile activities of fibroblasts were analyzed using fibroblast-populated collagen lattice (FPCL); images were obtained at 0, 24, and 48 h (*n* = 3). Control is untreated cells. Data are expressed as the means ± SD. *P < 0.05, **P < 0.01, and ***P ≤ 0.001, compared with control. NS, not significant; α-SMA, α-smooth muscle actin; ColI, collagen I; GMQ, 2-guanidine-4-methylquinazoline.

The healing process after skin injury includes three overlapping stages, inflammation, proliferation, and remodeling, and interactions between multiple cell types and the influence of cytokines are involved in the healing process [29]. In addition, the development of inflammation affects the efficiency and effectiveness of wound repair in the subsequent stages [30]. Macrophages are one of the essential inflammatory cell types in wound healing [31, 32]. Macrophages secrete cytokines, which are involved in tissue repair processes such as skin scarring [7, 33]. We thus hypothesized that ASIC3 might mediate fibroblast-to-myofibroblast differentiation indirectly through macrophages. We next performed co-culture experiments. We first activated the fibroblast ASIC3 receptor as well as overexpressed the ASIC3 receptor (ASIC3-OE) in media containing GMQ or pH 5.0. After 24 h, the fibroblasts and macrophages were co-cultured for 48 h (Fig. 3A). We then measured the expressions of α-SMA and COL-I produced by myofibroblasts or activated fibroblasts. Immunofluorescence staining showed that α-SMA and COL-I were increased in activated fibroblasts after ASIC3 activation by acidic solution (pH 5.0) or the GMQ ASIC3 activator; co-treatment of acidic solution (pH 5.0) or GMQ with ASIC3 overexpression resulted in more pronounced effects (Fig. 3B). Western blot results of α-SMA (Fig. 3C) and qPCR results of α-SMA and COL-I mRNA were consistent with these findings (Fig. 3D, E). These results indicate that when macrophages were in direct contact with fibroblasts in co-culture, ASIC3 activation significantly induced myofibroblast differentiation.

The migratory and contractile capability of fibroblasts is essential to the pathogenesis of hypertrophic scar [34]. We thus examined the effects of ASIC3 activation on fibroblasts under co-culture conditions. In wound-healing and collagen gel contraction assays, ASIC3 activation not only significantly increased the migration of fibroblasts (Fig. 3F, G) but it also induced their contractile capability (Fig. 3H, I). Furthermore, the migratory and contractile capacities of fibroblasts were increased by ASIC3 overexpression in these cells. Together, these data indicate that ASIC3 activation-induced fibroblast-to-myofibroblast differentiation indirectly through effects on macrophages.

### ASIC3-mediated macrophage M2 polarization promotes fibroblast differentiation in vitro and in vivo

**Fig. 4:**
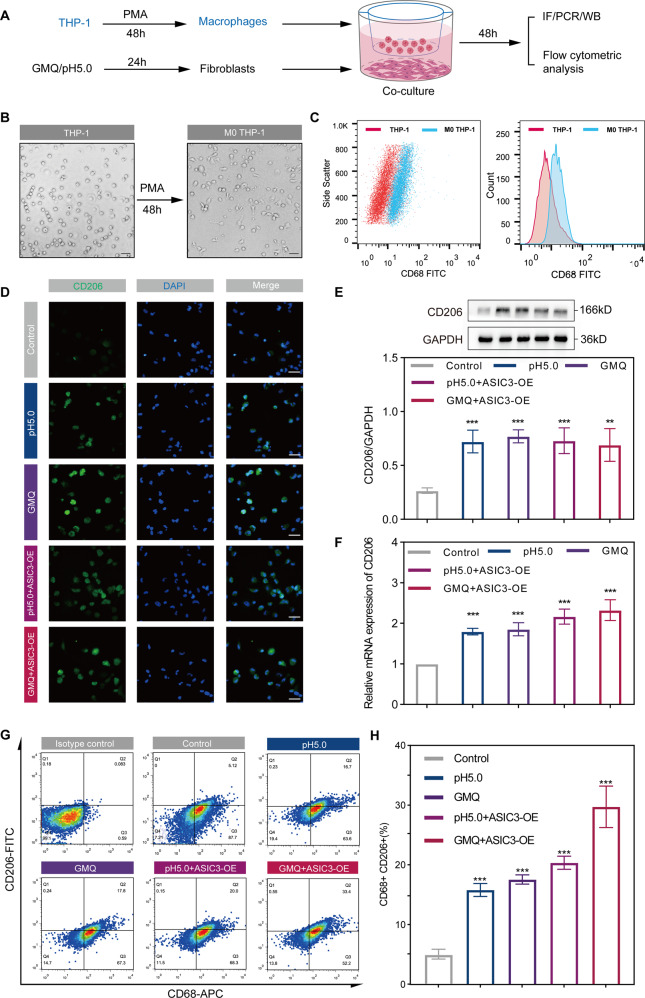
Activation of ASIC3 induces polarization of M0 macrophages to M2 macrophages in vitro. **A** Experimental design of fibroblast and macrophage indirect co-culture assay. **B** Representative images of THP-1 cells and 100 ng/ml PMA-derived M0 macrophages. **C** Flow cytometric analysis of CD68 in THP-1 cells and 100 ng/ml PMA-derived M0 macrophages. **D** Immunofluorescence staining of CD206 (green) in experimental and control macrophages after 48 h of co-cultivation. Scale bars, 100 μm. **E**, **F** Western blot and RT-qPCR analysis of CD206 protein levels and relative mRNA levels expression in the indicated groups (*n* = 3). GAPDH served as a loading control. **G** Expressions of CD68 and CD206 were determined by flow cytometry. **H** Quantification of the flow cytometry assay results (n = 3). Data are expressed as the means ± SD. *P < 0.05, **P < 0.01, and ***P ≤ 0.001. THP-1, human acute monocytic leukemia cell line; PMA, phorbol 12-myrisate 13-acetate; APC, allophycocyanin; FITC, fluorescein-isothiocyanate; GMQ, 2-guanidine-4-methylquinazoline.

Macrophages, an essential inflammatory cell type, exhibit many functions in wound repair [7, 35]. The different phenotypes of macrophages have been confirmed to be closely related to wound remodeling, scar formation, and fibrotic diseases [7, 33]. Macrophages can be activated into two distinct phenotypes, M1 and M2 [36]. Our data indicated that activation of ASIC3 indirectly promotes the differentiation of fibroblasts into myofibroblasts through effects in macrophages. We thus explored the impact of activation of fibroblast ASIC3 receptors on macrophages. THP-1 is a human immortalized monocyte-like cell line that is often used for in vitro studies of monocyte differentiation and function [37]. To explore the influence of fibroblasts on macrophages, we used PMA to induce THP-1 cells to differentiate into macrophages [38]. THP-1-derived macrophages and ASIC3 receptor-activated fibroblasts were co-cultured in Transwells (Fig. 4A). PMA-differentiated human THP-1 monocytes (M0 macrophages) that became adherent were observed under an optical microscope (Fig. 4B). Flow cytometry confirmed the monocyte-to-macrophage differentiation that was characterized by increased CD68 expression (Fig. 4C).

Previous studies have shown that macrophages undergo morphological changes in response to various different molecular signals [39]. When stimulated with the M1 polarization inducer LPS and IFN-γ, the macrophages are round; IL-4 stimulation, the M2 polarization inducer, has a prolonged effect on the macrophages [40]. Elongated macrophages tend to polarize into M2 macrophages and inhibit M1 polarization [41]. We found that compared with the control group, the other treatment groups showed more elongated macrophages (Figure S3A). Notably, immunofluorescence staining showed that the treated macrophages expressed higher CD206 expression (Fig. 4D), indicating that the polarization state of macrophages is related to the M2 phenotype. We further found that CD206 was significantly increased, and ASIC3 was overexpressed more significantly (Fig. 4E, F). These findings indicate that ASIC3 activation induces M2 macrophage polarization.

We then evaluated the macrophage surface markers CD68 and M1 and M2 macrophage surface markers CD86 and CD206 by flow cytometry. The ASIC3 agonist GMQ and pH 5.0 treatment increased the proportion of CD68 + CD206+ cells, from 5.12% to 16.7% and 17.8%, respectively (Fig. 4G, H). The proportions of CD68 + CD206+ cells in the pH 5.0 + ASIC3-OE and GMQ + ASIC3-OE treatment groups were increased to 20.0% and 33.4%, respectively, compared with the control group. We did not observe any differences in expression of the CD86 M1 macrophage surface marker between experimental groups under the same experimental conditions (Fig. S3B). This confirms that ASIC3 activation effectively drives the polarization of M2 macrophages in vitro.

**Fig. 5:**
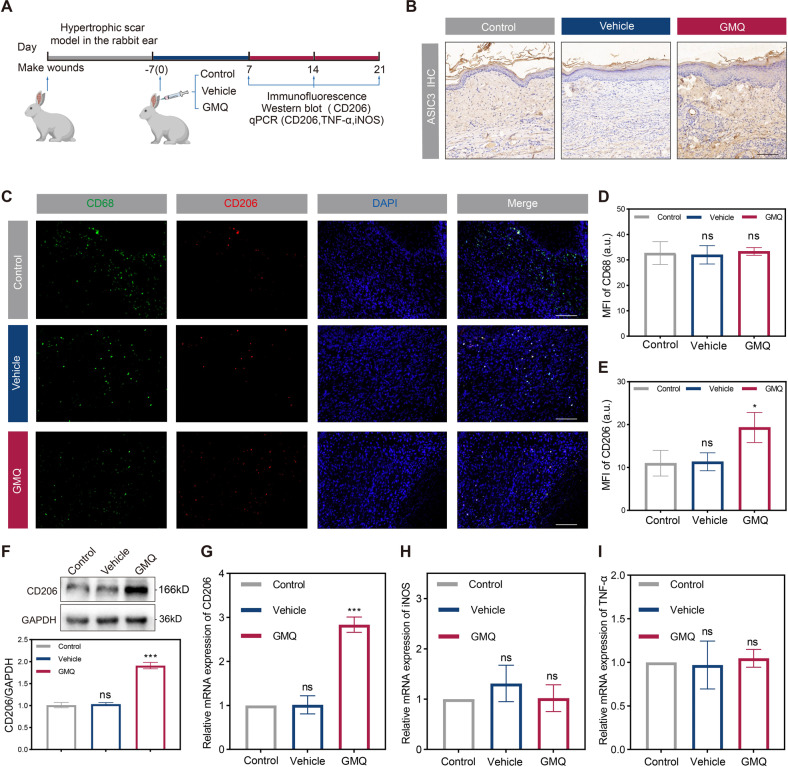
Activation of ASIC3 induces polarization of macrophages to M2 phenotype in vivo. **A** Schematic diagram of establishing rabbit ear hypertrophic scar model and experimental plan. **B** Immunohistochemistry analysis of ASIC3 expression in rabbit ear tissue 21 days after wounding. Scale bars, 100 μm. **C** Immunofluorescence staining of rabbit ear tissue 21 days after wounding showing the total (CD68+, green) and M2 subtype (CD206+, red) macrophages after treatment by PBS, DMSO, or GMQ. Scale bars, 100 μm. **D**, **E** MFI quantification of CD68 and CD206 in (C) (*n* = 3). **F**, **G** Western blot and RT-qPCR analysis of rabbit ear tissue 21 days after wounding. Total CD206 protein levels and relative mRNA levels expression in control, vehicle, and GMQ groups (*n* = 3). **H**, **I** Relative mRNA expressions of M1 macrophage markers (iNOS, TNF-α) as evaluated by RT-qPCR (*n* = 3). Data are expressed as the means ± SD. *P < 0.05, ***P ≤ 0.001. NS, not significant. iNOS, inducible nitric oxide synthase; TNF-α, tumor necrosis factor alpha.

Macrophages are the primary inflammatory cells in the process of wound healing and scar formation [7, 32]. There have been many studies on the polarization of their cell phenotypes [33]. In vitro co-culture system experiments confirmed that the activation of ASIC3 can significantly promote the M2 polarization of macrophages. We next explored whether activation of ASIC3 has the same effect on M2 polarization in vivo using a rabbit ear scar model with various treatments (Fig. 5A). Immunohistochemical experiments confirmed that the ASIC3 agonist GMQ increased ASIC3 expression (Fig. 5B).

We next performed immunofluorescence for CD206, the surface marker of M2 macrophages, and CD68, the surface marker of all subgroups of macrophages. As shown in Figure S3E, the groups had fewer CD206-positive macrophages at day 7. However, only the GMQ-treated group showed a significant increase in the number of CD206-positive macrophages at 14 and 21 days post-injury compared with the other groups (Fig. 5D, E, Figure S3F). These findings suggest that ASIC3 activation promotes M2 polarization in macrophages in vivo. We also found that the mRNA and protein expression levels of CD206 were significantly increased in the GMQ-treated group compared with the control group (Fig. 5F, G), but no significant differences were seen in the M1 markers iNOS and TNF-α (Fig. 5H, I). Together these data indicate that ASIC3 activation may promote scar formation by inducing M2 polarization and its influence on inflammation and fibrosis in the wound-healing stage.

### Activation of ASIC3 induces the release of M-CSF from fibroblasts

**Fig. 6:**
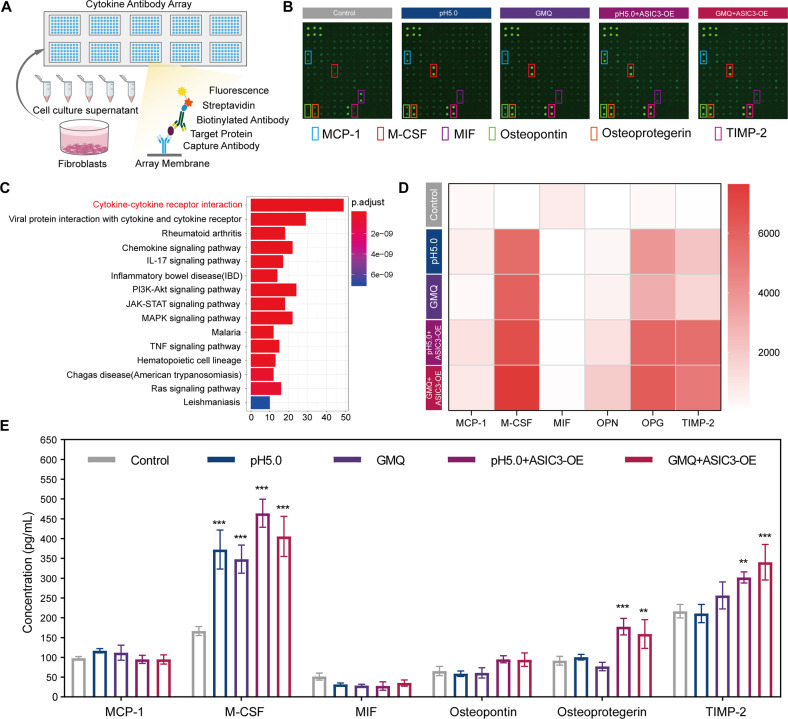
Activation of ASIC3 induces release of M-CSF from fibroblasts. **A** Experimental scheme of the cytokine antibody array. **B** The 80 cytokine antibody array was used to examine the expression of cytokines in cell supernatant. **C** Array results were analyzed by KEGG pathway enrichment analysis. **D** Semi-quantitative analysis of the expressions of MCP-1, M-CSF, MIF, OPN, OPG, and TIMP-2 presented by heat map. **E** MCP-1, M-CSF, MIF, OPN, OPG, and TIMP-2 expressions were determined by ELISA in cells treated as indicated for 48 h. Data are expressed as the means ± SD. *P < 0.05, **P < 0.01, and ***P ≤ 0.001, compared with controls. MCP-1, monocyte chemoattractant protein-1; M-CSF, macrophage colony-stimulating factor; MIF: macrophage migration inhibitory factor; OPN, osteopontin; OPG, osteoprotegerin; TIMP-2, tissue inhibitor of metalloproteinases 2; GMQ, 2-guanidine-4-methylquinazoline.

Previous studies have shown that ASIC3 is a critical molecule in the development of inflammatory pain [42, 43]. Our results suggest that ASIC3 induces the M2 polarization of macrophages. We speculate that one possible mechanism for the effects of ASICs is by impacting macrophage recruitment by regulating the production of secreted inflammatory cytokines. We conducted an inflammatory cytokine antibody array to investigate the potential impact of ASIC3 activation on the expression of 80 common cytokines/chemokines (Fig. 6A, B). Gene Ontology (GO) enrichment indicated that the top-ranked candidates were mainly involved in “cytokine-cytokine receptor interaction” (Fig. 6C). Semi-quantitative analysis of 80 cytokines and the top five cytokines with significant differences is displayed in a heat map (Fig. 6D, Figure S3D). MCP-1, M-CSF, MIF, OPN, OPG, and TIMP-2 expressions in treatment groups were significantly increased compared with levels in controls (fold change >1.5). As shown in Fig. 6D, M-CSF expression increased significantly, indicating M-CSF, which has roles various inflammatory-associated processes, was the most affected cytokine.

To confirm the above results, we used ELISA to detect the expression of MCP-1, M-CSF, MIF, OPN, OPG, and TIMP-2. Consistent with the results of the inflammatory cytokine antibody array, the expression of M-CSF showed the most significant increase (Fig. 6E). Previous research showed that the cytokine M-CSF plays a pivotal role in macrophage generation and function [44, 45]. Our results indicate that activation of ASIC3 promotes the increase of M-CSF secretion by fibroblasts, which leads to the polarization of M2 macrophages.

### ASIC3 mediates fibroblast M-CSF secretion through the PI3K/Akt pathway

**Fig. 7:**
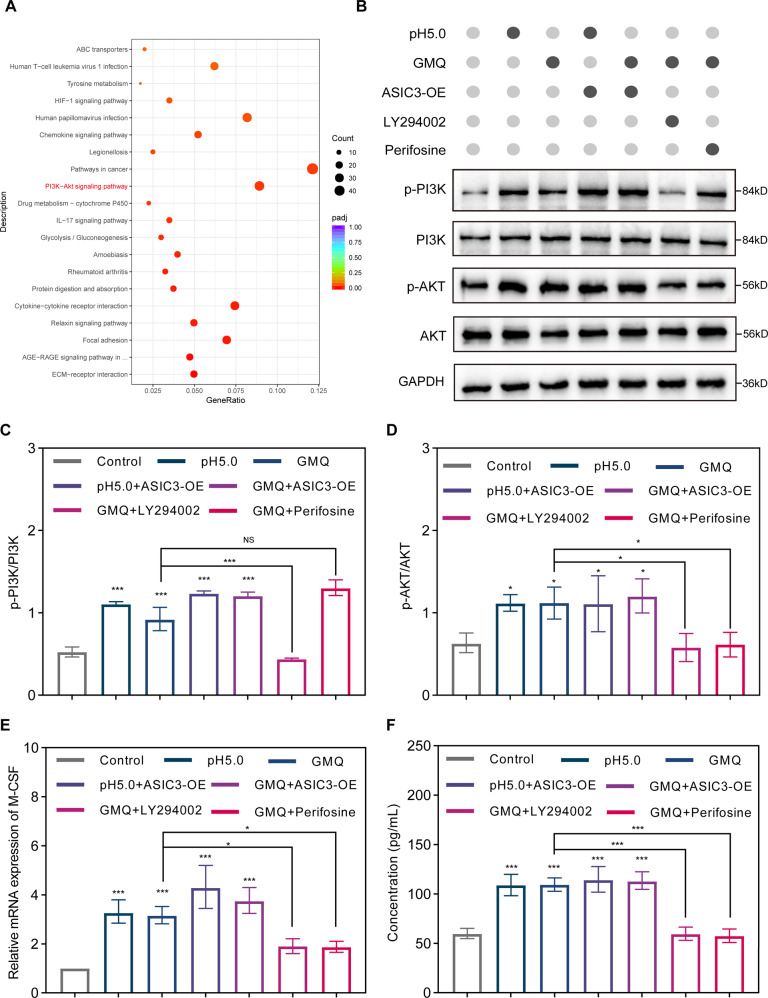
ASIC3 mediates induces the release of M-CSF from fibroblasts via the PI3K/AKT/M-CSF pathway. **A** KEGG pathway enrichment analysis. The top 20 KEGG pathways are shown. The size of the dot reflects the gene number; the dot color indicates the *q*-value**. B**–**D** Western blotting and quantitative analyses of the phosphorylation of PI3K and AKT in cells treated as indicated (*n* = 3). **E** RT-qPCR analysis of M-CSF mRNA in fibroblasts treated for 48 h with co-culture (*n* = 3). **F** ELISA of M-CSF expression in fibroblasts treated for 48 h with co-culture (*n* = 3). Data are expressed as the means ± SD. *P < 0.05, **P < 0.01, and ***P ≤ 0.001. PI3K, phosphoinositide 3-kinase; AKT, protein kinase B; p-PI3K, phospho-PI3K; p-AKT, phospho-AKT; LY294002, PI3K inhibitor; perifosine, AKT inhibitor; GAPDH, glyceraldehyde-3-phosphate dehydrogenase.

We next explored the potential mechanism underlying the regulatory effect of ASIC3 on fibroblasts. RNA sequencing analysis was performed on fibroblasts derived from human tissue primary culture and cells treated with GMQ for 24 h. A total of 9499 differentially expressed genes were identified (*P*-value <0.05, fold change > 0) (Figure S4A, B). KEGG enrichment analysis indicated that the PI3K/AKT pathway was specifically activated after GMQ treatment (Fig. 7A). GO enrichment analysis suggested that the differentially expressed genes are involved in ECM synthesis and wound healing (Figure S4C). We next performed western blotting to verify the RNA sequencing findings, and the results showed that p-PI3K and p-AKT were significantly upregulated after ASIC3 activation and increased to even higher levels with ASIC3 overexpression (Fig. 7B, C). However, qRT-PCR and ELISA showed that M-CSF mRNA expression and secretion were significantly reduced after PI3K/AKT signaling pathway blockade by inhibition of PI3K with LY294002 or inhibition of AKT by perifosine (Fig. 7D, E). Therefore, these findings suggest that ASIC3 activation may promote M-CSF transcription through the PI3K/AKT signaling pathway.

### ASIC3-mediated macrophage polarization and macrophage M2-derived TGF-β1 induces fibroblast-to-myofibroblast differentiation

**Fig. 8:**
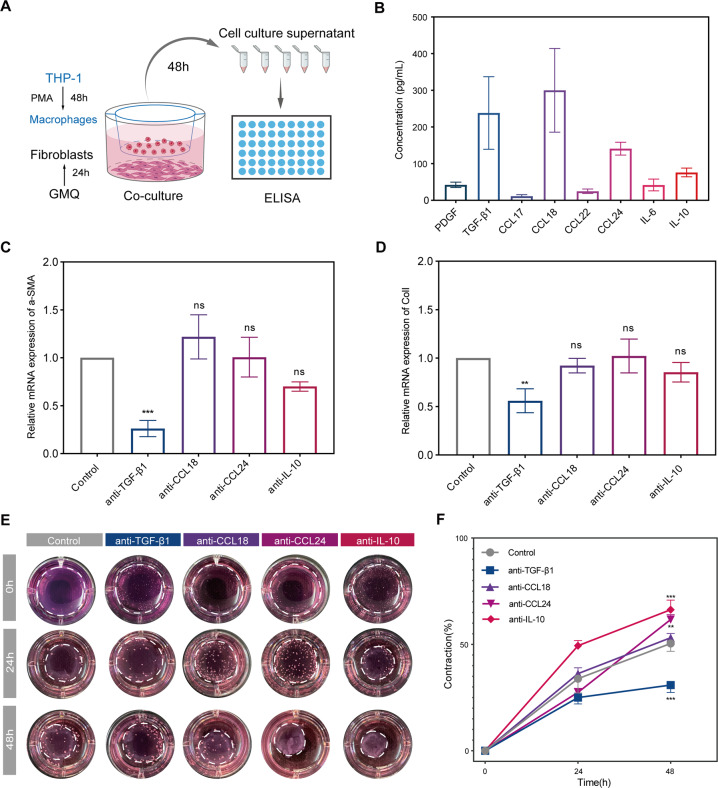
M2 macrophage-derived TGF-β1 induces fibroblast-to-myofibroblast differentiation. **A** Schematic diagram of the indirect co-culture cell model and experimental plan. **B** ELISA results of cytokines secreted by macrophages after ASIC3 activation by the GMQ ASIC3 activator (*n* = 3). **C**, **D** Fibroblasts were treated with neutralizing antibody and evaluated for α-SMA and collagen I expression (*n* = 3). **E**, **F** The contractile activities of fibroblasts treated as indicated were analyzed using fibroblast-populated collagen lattice contraction after 48 h of co-culture (*n* = 3). Data are expressed as the means ± SD. *P < 0.05, **P < 0.01, and ***P ≤ 0.001. NS, not significant; α-SMA, α-smooth muscle actin; ColI, collagen I; CCL18, C-C motif chemokine ligand 18; CCL24, C-C motif chemokine ligand 24; IL-10, interleukin 10; GMQ, 2-guanidine-4-methylquinazoline; THP-1, human acute monocytic leukemia cell line.

To explore the mechanism underlying the differentiation of fibroblasts to myofibroblasts by M2 macrophages, we performed ELISA to screen cytokines related to macrophages (Fig. 8A). Among the eight cytokines examined, TGF-β1, CCL18, CCL24, and IL-10 were the cytokines most predominantly secreted by macrophages after ASIC3 activation by the GMQ ASIC3 activator (Fig. 8B). We next used neutralizing antibodies to test the involvement of these factors in the differentiation of fibroblasts to myofibroblasts. Only anti-TGF-β1 treatment significantly alleviated the suppression of expressions of α-SMA and COL-I expression on fibroblasts (Fig. 8C, D). The other neutralizing antibodies had little effect on the expressions of α-SMA and COL-I expression.

**Fig. 9:**
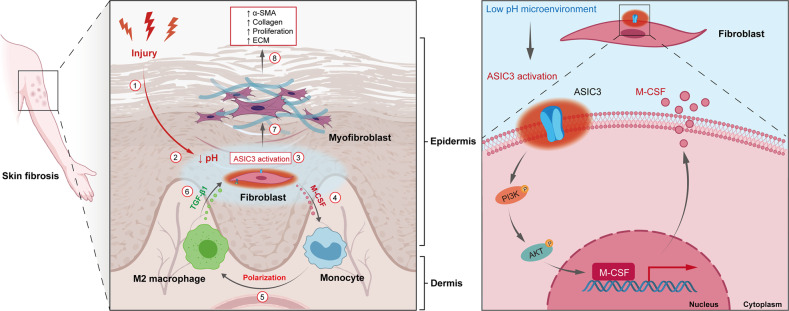
Schematic diagram of the ASIC3-M-CSF-M2 macrophage-positive feedback loop in skin fibrosis pathogenesis. Local injury leads to the formation of an acidic microenvironment, which activates fibroblast ASIC3 receptors and promotes the increase of M-CSF secretion through the PI3K/AKT signaling pathway, leading to the polarization of M2 macrophages and increasing the secretion of TGF-β1, which mediates fibroblasts to myofibroblast differentiation.

The fibroblast-populated collagen lattice (FPCL) experiment results further showed that anti-TGF-β1 treatment significantly inhibited the contraction of FPCL, which indirectly indicates inhibition of the differentiation of fibroblasts to myofibroblasts (Fig. 8E, F). These results suggest that the promotion of fibroblast differentiation to myofibroblasts by M2 macrophages is dependent on TGF-β1, implicating an ASIC3-M-CSF-M2 macrophage-positive feedback loop in myofibroblast activation (Fig. 9).

## Discussion and conclusion

Extracellular acidosis is a hallmark of inflammatory processes [46–48]. Previous studies mainly focused on the influence of ASICs on the intrinsic properties of injured cells. However, recent reports revealed that ASICs also shape interactions between injured cells and their associated stroma [13, 49, 50]. In this study, we describe a signaling pathway that regulates the dermis’ inflammatory environment and is mediated by ASIC3, which modulates the sequential recruitment and infiltration of macrophages into the inflammatory environment by its target, M-CSF. The recruited macrophages then enter into a paracrine loop with fibroblasts to enhance fibroblast differentiation into myofibroblasts.

ASIC3 participates in the development of several physiological and physiological processes. However, its roles in the pathogenesis of hypertrophic scars remain unclear. In this study, we found that ASIC3 expression was positively correlated with collagen deposition in hypertrophic scar and keloid. Activation of ASIC3 by GMQ and acidosis caused ECM deposition in the wound area, suggesting a promoting role of scar formation for ASIC3. The main pathological change underlying hypertrophic scar formation is the development of numerous myofibroblasts. We thus examined whether ASIC3 activation is involved in fibroblast-to-myofibroblast differentiation in vitro and in vivo. Through α-SMA expression analysis, we found that ASIC3 activation was not directly involved in ECM protein deposition. A recent report revealed that ASIC1a and ASIC3 contribute to nucleus pulposus cell inflammation that is stimulated by extracellular lactate [51]. This observation, which is consistent with our research results, indicates that ASIC3 activation may cause the release of M-CSF by fibroblasts. ASIC3 may indirectly regulate ECM remodeling via cell inflammation.

The THP-1 acute monocytic leukemia cell line is frequently used in various fields of research. After differentiation of THP-1 cells using PMA or other stimuli, a macrophage phenotype is obtained that mimics native human macrophages but differs from autologous cells. Whether activation of ASIC3 specifically promotes the polarization of circulating or tissue-resident macrophages is not clear and will be explored in our follow-up studies.

M-CSF, which signals through its receptor, stimulates monocyte viability [44, 45]. However, the role of M-CSF in scar development remains largely unknown. In vitro and in vivo studies revealed that the effects of M-CSF on fibroblast differentiation into myofibroblasts are mediated by the recruitment and infiltration of M2 macrophages. M-CSF was confirmed to have a central role in M2 macrophage polarization, and we demonstrated that activation of ASIC3 induces the release of M-CSF from fibroblasts, causing macrophage M2 polarization. Together, these findings indicate that M2 polarization is mediated by M-CSF in fibroblasts. Further investigation confirmed that activation of PI3K/AKT signaling pathways in fibroblast may play a crucial role in ASIC3-mediated effects.

TGF-β1 is a master profibrotic cytokine that is produced by immune cells and plays a central role in fibrosis by promoting the differentiation of fibroblasts into myofibroblasts [52, 53]. Our results confirmed that ASIC3 activation had no effects on TGF-β1 expression in fibroblasts both in vitro and in vivo. However, when the macrophages were in direct contact with fibroblasts via co-culture, ASIC3 activation significantly increased TGF-β1 expression. Moreover, anti-TGF-β1 treatment significantly alleviated the suppression of α-SMA expression on fibroblasts by M2 macrophages. Thus, we conclude that the promotion of fibroblasts differentiate to myofibroblasts on fibroblasts by M2 macrophages is dependent on TGF-β1, implicating an ASIC3-M-CSF-M2 macrophage-positive feedback loop in myofibroblast activation.

Our findings demonstrate a role for ASIC3 in the development of skin fibrosis, indicating that ASIC3 is a promising therapeutic target. Our study confirmed that ASIC3 expression was increased in disease-derived dermal fibrosing tissue in hypertrophic scar and keloid patients. Furthermore, we found that ASIC3 expression was positively correlated with collagen deposition in skin fibrosis. ASIC3 acts as a pH sensor in cells and may have a broader role in cell physiology and pathology. Therefore, whether ASIC3 regulates inflammation and other potential mechanisms of skin fibrosis remains to be further studied.

Taken together, our results describe an ASIC3-M-CSF-M2 macrophage-positive feedback loop that modulates fibroblast-to-myofibroblast differentiation. Thus, ASIC3 may provide a molecular basis for sensing acid stress in skin fibrosis pathogenesis. Targeting ASIC3 may be a novel therapeutic strategy for skin fibrosis.

## Supplementary information

Supplementary material

Supplementary Figure 1

Supplementary Figure 2

Supplementary Figure 3

Supplementary Figure 4

Original Data File

checklist

## Data Availability

The original contributions presented in the study are publicly available. This data can be found here: About RNA sequences may view GSE186739 study at https://www.ncbi.nlm.nih.gov/geo/query/acc.cgi?acc=GSE186739.
