# Supplementary material for: The ASIC3-M-CSF-M2 macrophage-positive feedback loop modulates fibroblast-to-myofibroblast differentiation in skin fibrosis pathogenesis
Source: Cell Death Dis. 2022 Jun 6;13(6):527. doi: 10.1038/s41419-022-04981-9 (PMC9167818; doi:10.1038/s41419-022-04981-9)
Supplement: Supplementary file 1 — Supplementary material [file 41419_2022_4981_MOESM1_ESM.doc]

**Supplementary Material**

**Supplementary Figure 1.**

**(A)** RT-qPCR analysis of ASIC1a, ASIC2a, ASIC3, and ASIC4 mRNAs in normal skin, hypertrophic scar and keloid (n = 3). **(B)** RT-qPCR of ASIC3 mRNA in fibroblasts derived from normal skin, hypertrophic scar tissue and keloid (n = 3). **(C)** CCK8 assay of the proliferation of fibroblasts treated by different concentrations of GMQ (n = 3). **(D)** Correlation analysis between ASIC3 and COL-I expression in hypertrophic scar tissue. R^2^ = 0.9007, P = 0.004 (n = 10). **(E)** Correlation analysis between ASIC3 and COL-I expression in keloid tissue. R^2^ = 0.8236, P = 0.034 (n = 10). **(F)** Fibroblasts were infected with lentivirus overexpressing ASIC3 or control vector. Total RNA was isolated after 48 h of infection. RT-qPCR was performed to detect ASIC3 mRNA expression in fibroblasts (n = 3). **(G)** Total proteins were extracted after 48 h of infection. Western blot analysis was performed to detect the expression of ASIC3 in fibroblasts. GAPDH expression was used as a loading control (n=3). **(H)** Immunofluorescence analysis of ASIC3 expression in cells infected with ASIC3-overexpressing lentivirus. Scale bars, 100 μm. Data are expressed as the means ± SD. *P<0.05, **P<0.01 and ***P≤0.001 compared with control. HS, hypertrophic scar; ASIC3-OE, ASIC3-overexpression; ColI, collagen I; DAPI, 4′,6-diamidino-2-phenylindole.

**Supplementary Figure 2.**

**(A)** Schematic diagram of cell culture and experimental design. **(B–C)** α-SMA and Collagen I mRNA expressions in the indicated groups were detected by RT-qPCR (n=3). **(D–F)** Western blot analysis of α-SMA and collagen I revealed no significant differences in the experimental groups compared with the control (n=3). **(G–H)** Wound healing assays of fibroblast migration at 12 or 24 h after wounding (n = 3). Scale bars, 100 μm. Data are expressed as the means ± SD. NS, not significant. α-SMA, α-smooth muscle actin; COL-I, collagen I; ASIC3-OE, ASIC3-overexpression.

**Supplementary Figure 3.**

1. Cell morphological change of THP-1 cells induced by 100 ng/ml PMA for 48 h of co-culture. Red arrowheads indicate elongated macrophages. Scale bars, 50 μm. **(B)** Histograms of CD86 expression at 48 h of co-culture by flow cytometric analysis; no significant differences were detected in experimental groups compared with the control. **(C)** Method for quantifying the scar elevation index (SEI); the SEI is the ratio of the total dermal area (X+Y), including the newly formed hypertrophied dermis, to the area of the old dermis (Y). **(D)** The expression of 80 cytokines was semi-quantitatively analyzed by fluorescence intensity. **(E)** Immunofluorescence analysis at 7 days and 14 days after wounding in rabbit ear tissue sections. Total (CD68+, green) and M2 subtype (CD206+, red) macrophages in the indicated groups (n=3).Scale bars, 100 μm. APC, allophycocyanin; ASIC3-OE, ASIC3-overexpression.

**Supplementary Figure 4.**

**(A)** Volcano plot for DEGs. The green and red dots indicate the significantly downregulated and upregulated genes, respectively; the blue dots represent the genes without differential expression. DEGs were identified by log 2 (fold-change) value >0 and P<0.05. **(B)** Heat map of cluster results for all genes. **(C)** The molecular function, biological process, and cellular component of DEGs were analyzed by Gene Ontology enrichment analysis; C, control; G, GMQ; BP, biological process; CC, cellular component; MF, molecular function.

**Supplementary Tables**

**Supplementary Table 1.** Characteristics of cases with human hypertrophic scars

| **No.** | **Sex** | **Age (years)** | **Biopsy site** | **Time after wounding** | **Type of wound** | **Wound size**  **(length × width)** |
| --- | --- | --- | --- | --- | --- | --- |
| 1 | Female | 25 | Abdomen | 4 years | Surgery | 10 cm × 1 cm |
| 2 | Female | 18 | Left-thigh | 2 years | Burn | 4.5 cm × 5.5 cm |
| 3 | Male | 23 | Right-foot | 5 years | Surgery | 2 cm × 8 cm |
| 4 | Male | 34 | Left-arm | 3 years | Trauma | 4 cm × 3 cm |
| 5 | Female | 22 | Clavicle | 1 years | Trauma | 1.5 cm × 1 cm |
| 6 | Male | 41 | Right-arm | 1 years | Burn | 7.5 cm × 3.5 cm |
| 7 | Female | 28 | Forehead | 7 years | Trauma | 1.5 cm × 1.5 cm |
| 8 | Female | 36 | Clavicle | 10 mo | Burn | 2.5 cm×0.5 cm |
| 9 | Male | 26 | Left-arm | 4 years | Surgery | 6 cm × 4 cm |
| 10 | Male | 42 | Right-foot | 6 years | Trauma | 9 cm × 0.5 cm |

**Supplementary Table 2. Characteristics of cases with human keloids**

| **No.** | **Sex** | **Age (years)** | **Biopsy site** | **Time after wounding** | **Type of wound** | **Wound size**  **(length × width)** |
| --- | --- | --- | --- | --- | --- | --- |
| 1 | Male | 28 | Sternum | 4 years | Surgery | 5 cm × 3 cm |
| 2 | Female | 21 | Right ear | 2 years | Piercing | 0.5 cm × 0.5 cm |
| 3 | Male | 21 | Forehead | 1 years | Burn | 4.5 cm × 1.5 cm |
| 4 | Female | 18 | Clavicle | 2 years | Burn | 7 cm × 4 cm |
| 5 | Female | 25 | Left foot | 4 years | Trauma | 6 cm × 1.5 cm |
| 6 | Male | 38 | Left shoulder | 2 years | Trauma | 7.5 cm × 1.5 cm |
| 7 | Female | 26 | Face | 4 years | Trauma | 3.5 cm × 1 cm |
| 8 | Female | 31 | Left thigh | 7 years | Burn | 10 cm×2.5 cm |
| 9 | Male | 19 | Right arm | 3 years | Surgery | 3 cm × 1.5 cm |
| 10 | Male | 27 | Upper-lip | 1 years | Surgery | 1 cm × 0.5 cm |

**Supplementary Table 3.** Sequence of primers used in quantitative RT-PCR

| **Gene** | **Forward primers (5'-3')** | **Reverse primers (5'-3')** | |
| --- | --- | --- | --- |
| ASIC3 | ACCTTCCTCTACCAGGTGGC | | TGGTGAAGTTCTCAGGCCCA |
| ASIC1α | CATGCTGCTCTCCTGCCACTTC | | GTTTCTCACCCTTTCTTGCCCTCTG |
| ASIC2α | GGCTGGAGATCATGCTGGACATTC | | CTTGGATGAAAGGTGGCTCAGACTG |
| ASIC4 | GCCTGGAGATCATGCTGGACATC | | CACCCGAATACCTGCCTCAAACG |
| α-SMA | CTCTGGACGCACAACTGGCATC | | GGCATGGGGCAAGGCATAGC |
| Collagen I | TGGCAAAGAAGGCGGCAAAGG | | AGGAGCACCAGCAGGACCATC |
| CD206 | TTCAGTGGACCATCGAGGAAGAGG | | ATGGCAACACACCCTGGCTTTC |
| iNOS | CAGGGTGGAAGCGGTAACAAAGG | | CCTGCTTGGTGGCGAAGATGAG |
| TNF-α | AGCCCTGGTATGAGCCCATCTATC | | TCCCAAAGTAGACCTGCCCAGAC |
| M-CSF | CCCTCCACCCTCTCTGCTCAG | | CTTCACTTGCTGGTCCTCCTTCTG |
| GAPDH | AATCCCATCACCATCTTCCA | | TGGACTCCACGACGTACTCA |

**Supplementary Table 4.** The expression of 80 cytokines was examined by a cytokine antibody array.

|  | A | B | C | D | E | F | G | H | I | J | K | L | M |
| --- | --- | --- | --- | --- | --- | --- | --- | --- | --- | --- | --- | --- | --- |
| 1 | POS1 | POS2 | POS3 | NEG | NEG | NEG | E N A -78 | CSF3 | CSF2 | GRO | GRO-α | I-309 | IL-1α |
| 2 | POS1 | POS2 | POS3 | NEG | NEG | NEG | E N A -78 | CSF3 | CSF2 | GRO | GRO-α | I-309 | IL-1α |
| 3 | IL-1β | IL-2 | IL-3 | IL-4 | IL-5 | IL-6 | IL-7 | IL-8 | IL-10 | IL-12 p40/p70 | IL-13 | IL-15 | IFN-γ |
| 4 | IL-1β | IL-2 | IL-3 | IL-4 | IL-5 | IL-6 | IL-7 | IL-8 | IL-10 | IL-12 p40/p70 | IL-13 | IL-15 | IFN-γ |
| 5 | MCP-1 | MCP-2 | MCP-3 | ANG | MDC | MIG | MIP-1β | MIP-1δ | RANTES | SCF | SDF-1 | TARC | TGF-β1 |
| 6 | MCP-1 | MCP-2 | MCP-3 | ANG | MDC | MIG | MIP-1β | MIP-1δ | RANTES | SCF | SDF-1 | TARC | TGF-β1 |
| 7 | TNF-α | TNF-β | EGF | IGF-I | CSF1 | OSM | THPO | VEGF-A | PDGF- BB | LEPTIN | BDNF | BLC | CKβ 8-1 |
| 8 | TNF-α | TNF-β | EGF | IGF-I | CSF1 | OSM | THPO | VEGF-A | PDGF- BB | LEPTIN | BDNF | BLC | CKβ 8-1 |
| 9 | CCL11 | CCL24 | CCL26 | FGF4 | FGF6 | FGF7 | FGF9 | Flt-3LG | CX3CL1 | GCP-2 | GDNF | HGF | IGFPBP1 |
| 10 | CCL11 | CCL24 | CCL26 | FGF4 | FGF6 | FGF7 | FGF9 | Flt-3LG | CX3CL1 | GCP-2 | GDNF | HGF | IGFPBP1 |
| 11 | IGFBP2 | IGFBP3 | IGFBP4 | IL-16 | IP-10 | LIF | LIGHT | MCP4 | MIF | MIP-3α | NAP-2 | NT-3 | NT-4 |
| 12 | IGFBP2 | IGFBP3 | IGFBP4 | IL-16 | IP-10 | LIF | LIGHT | MCP4 | MIF | MIP-3α | NAP-2 | NT-3 | NT-4 |
| 13 | OPN | OPG | PARC | PLGF | TGF-β2 | TGF-β3 | TIMP1 | TIMP2 | NEG | NEG | NEG | NEG | NEG |
| 14 | OPN | OPG | PARC | PLGF | TGF-β2 | TGF-β3 | TIMP1 | TIMP2 | NEG | NEG | NEG | NEG | NEG |

CSF1 = M-CSF, CSF2 = GM-CSF, CSF3 = G-CSF, ANG = Angiogenin, OSM = Oncostatin M, THPO = Thrombopoietin, OPN = Osteopontin, CKβ8-1 = CCL23,

CCL11 = Eotaxin, CCL24 = Eotaxin-2, CCL26 = Eotaxin-3, Flt-3 LG = Flt-3 Ligand,

CX3CL1 = Fractalkine, OPG = Osteoprotegerin, IL-12 reacts with both IL-12p40 and IL-12p70, GRO reacts with CXCL1, CXCL2 and CXCL3 (GRO alpha, beta and gamma, respectively), GRO alpha reacts only with CXCL1, VEGF-A reacts with VEGF-165 and VEGF-121, and TGF-β1 reacts only with the active form of TGF-β1.
