# Supplementary figures and images for: The ASIC3-M-CSF-M2 macrophage-positive feedback loop modulates fibroblast-to-myofibroblast differentiation in skin fibrosis pathogenesis
Source: Cell Death Dis. 2022 Jun 6;13(6):527. doi: 10.1038/s41419-022-04981-9 (PMC9167818; doi:10.1038/s41419-022-04981-9)

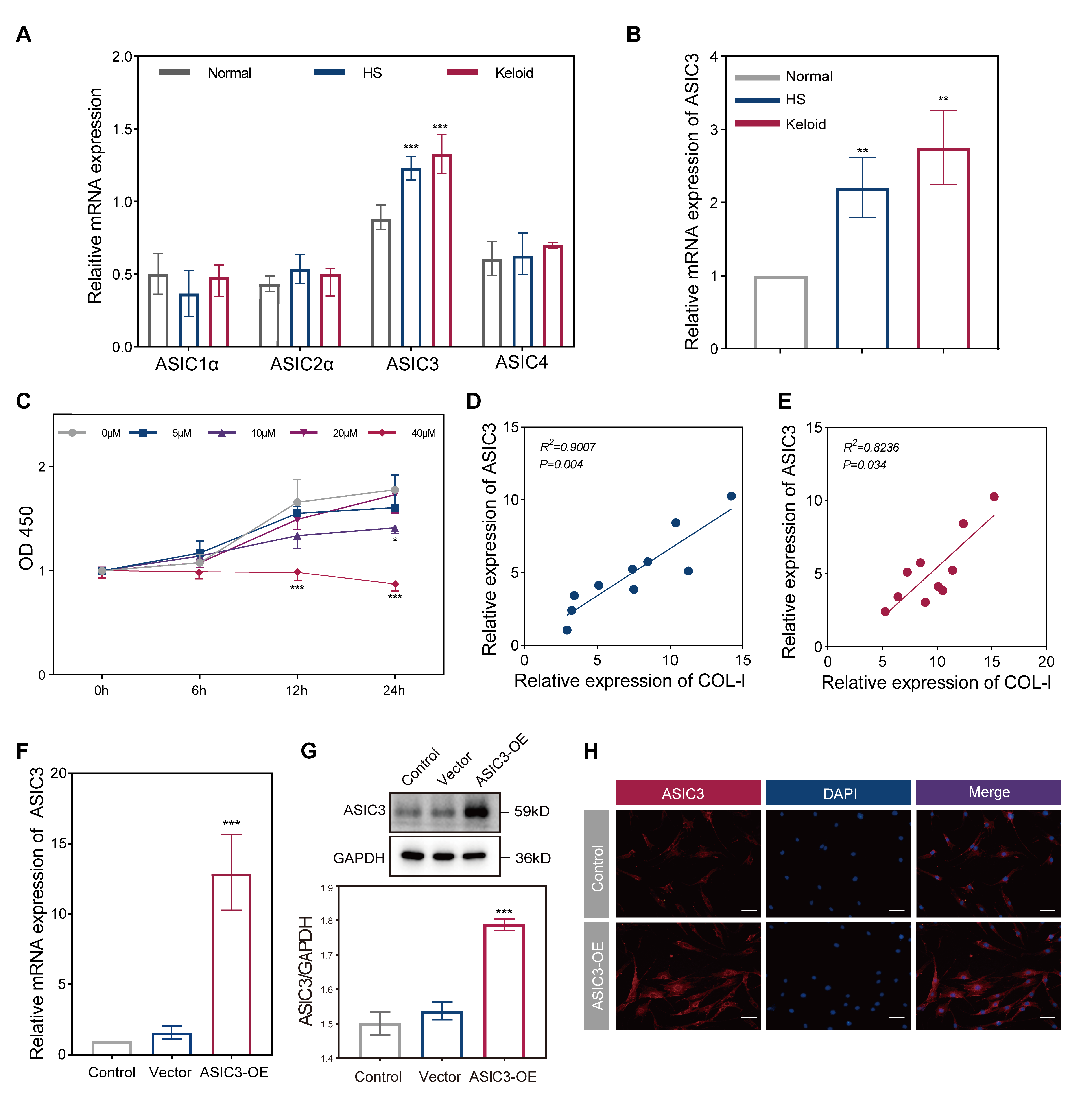

Supplement: Supplementary file 2 — Supplementary Figure 1 [file 41419_2022_4981_MOESM2_ESM.tif]

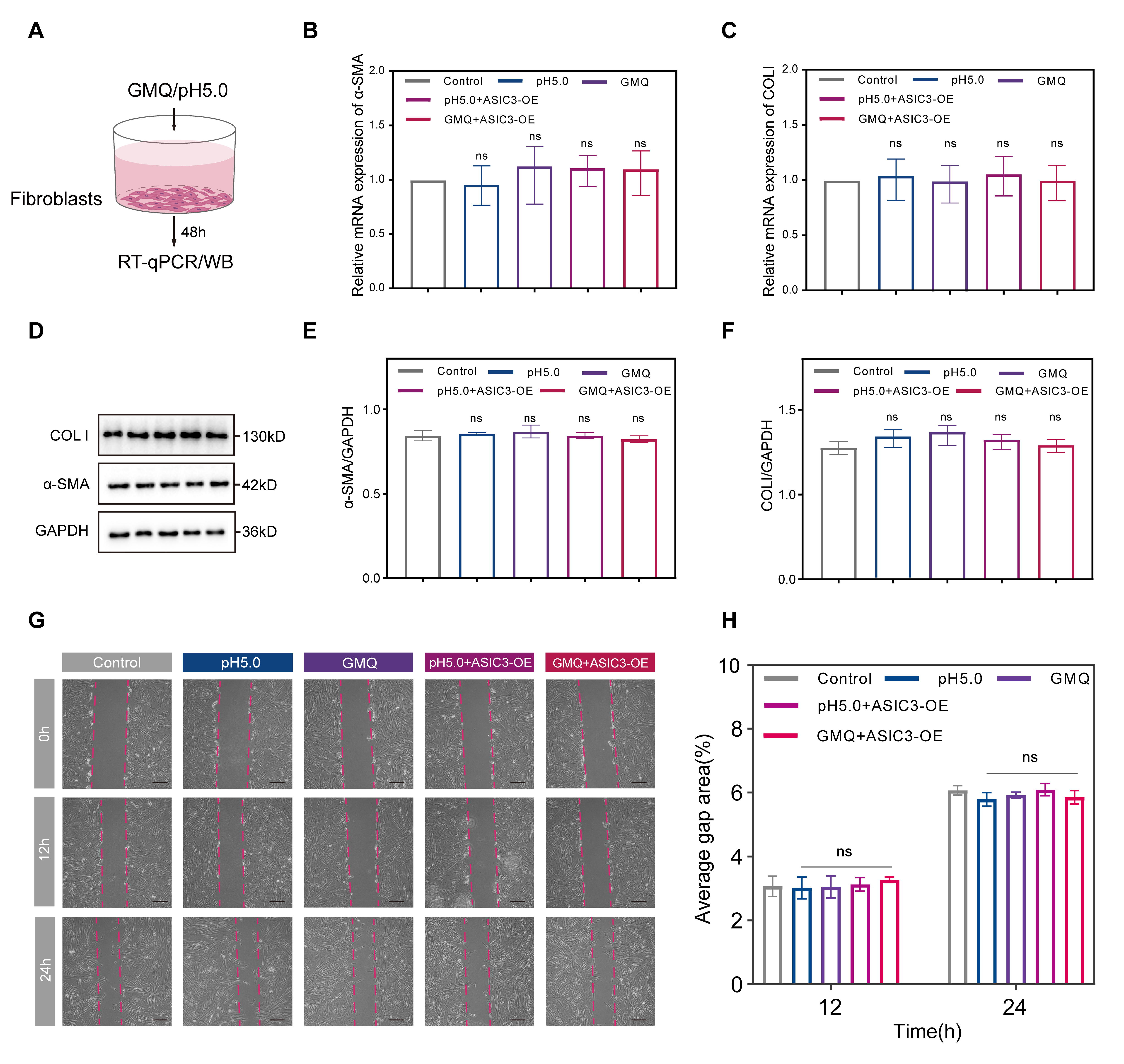

Supplement: Supplementary file 3 — Supplementary Figure 2 [file 41419_2022_4981_MOESM3_ESM.tif]

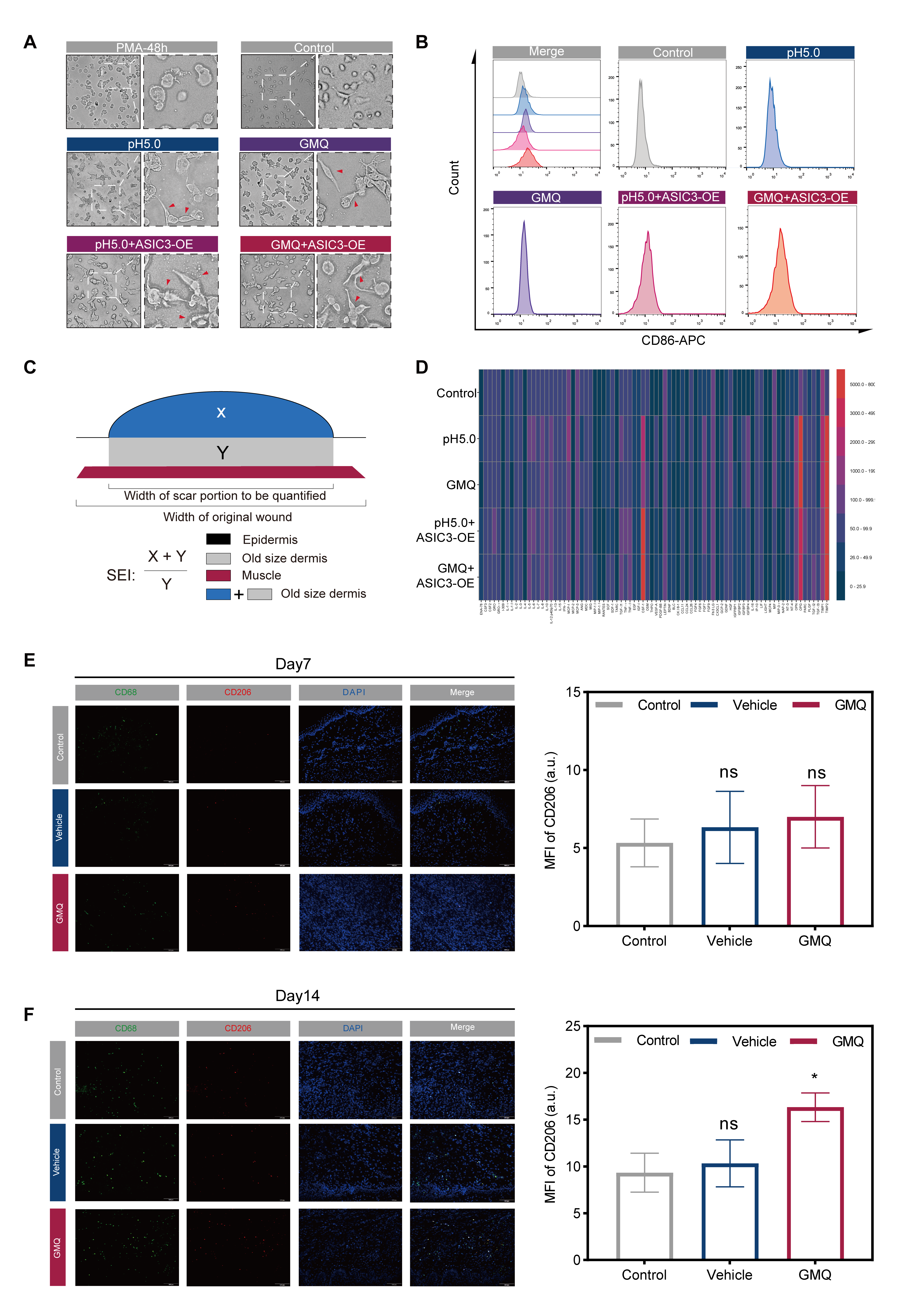

Supplement: Supplementary file 4 — Supplementary Figure 3 [file 41419_2022_4981_MOESM4_ESM.tif]

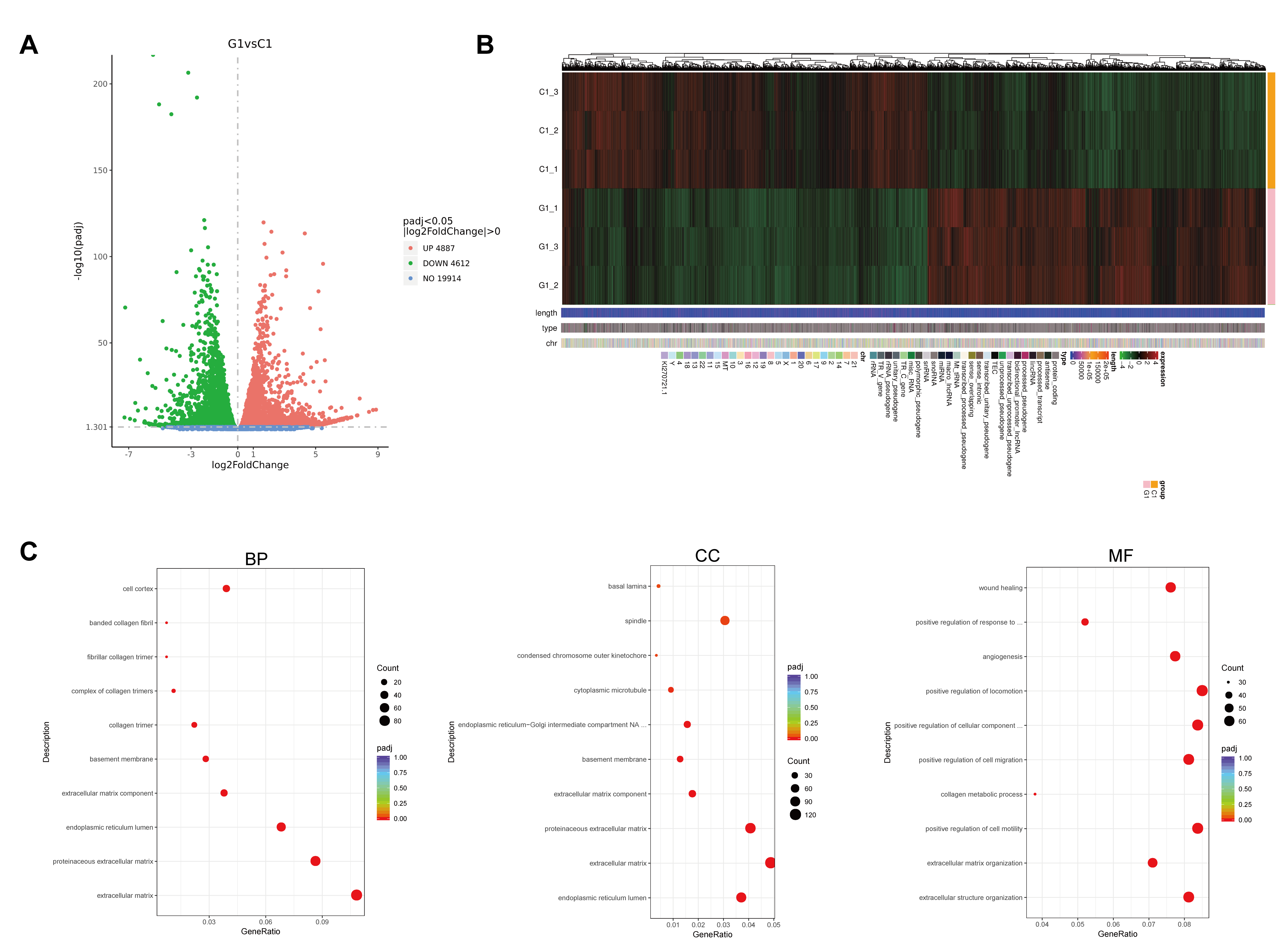

Supplement: Supplementary file 5 — Supplementary Figure 4 [file 41419_2022_4981_MOESM5_ESM.tif]
