# Supplementary material for: The ASIC3-M-CSF-M2 macrophage-positive feedback loop modulates fibroblast-to-myofibroblast differentiation in skin fibrosis pathogenesis
Source: Cell Death Dis. 2022 Jun 6;13(6):527. doi: 10.1038/s41419-022-04981-9 (PMC9167818; doi:10.1038/s41419-022-04981-9)

**Source data**

Images of the original western blots for review

Figure 1. ASIC3 is highly expressed in human hypertrophic scar/keloid tissues and human primary cells.


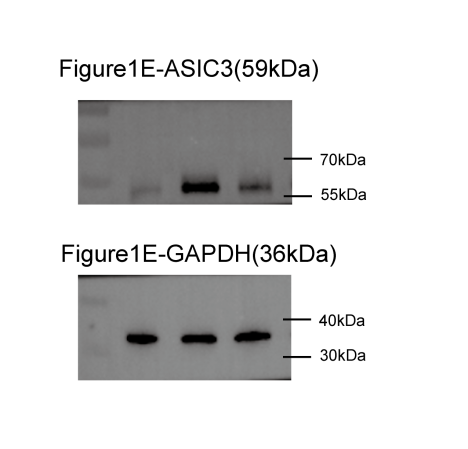


Figure 2. ASIC3 promotes scar formation in vivo.


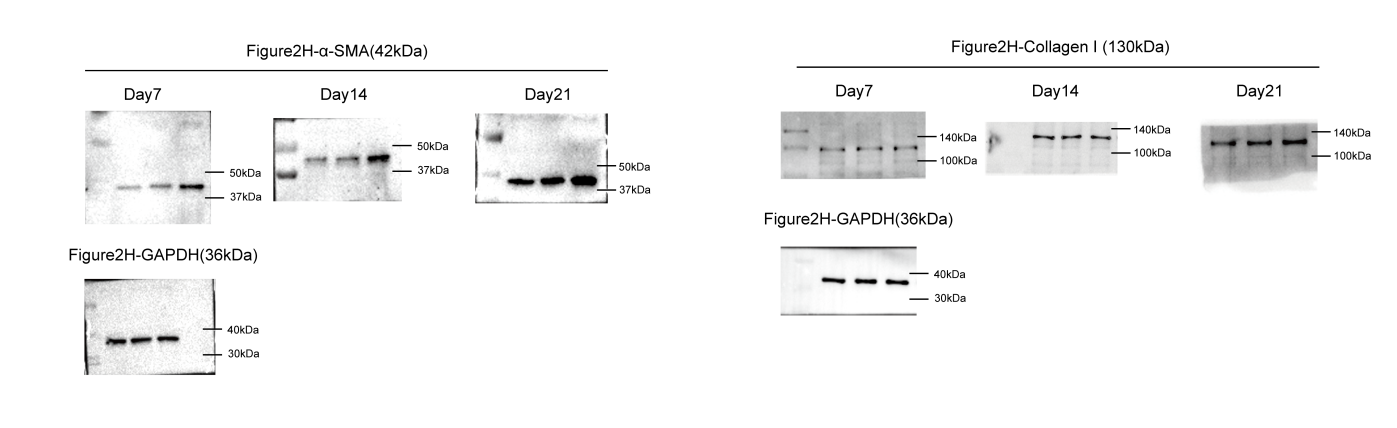


Figure 3. Activation of ASIC3 promotes fibroblast-to-myofibroblast differentiation through a process requiring macrophages.


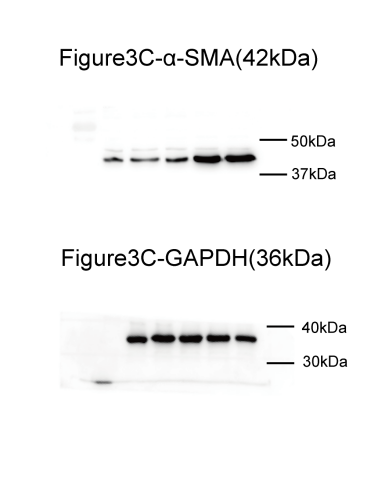


Figure 4. Activation of ASIC3 induces polarization of M0 macrophages to M2 macrophages in vitro.


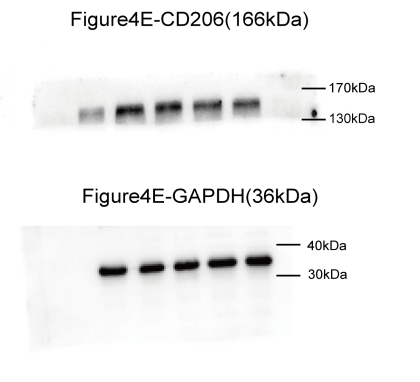


Figure 5. Activation of ASIC3 induce polarization of macrophages to M2 phenotype in vivo.


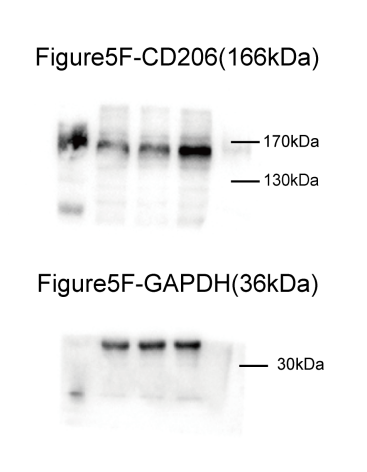


Figure 7. ASIC3 mediates induces the release of M-CSF from fibroblasts via the PI3K/AKT/M-CSF pathway.


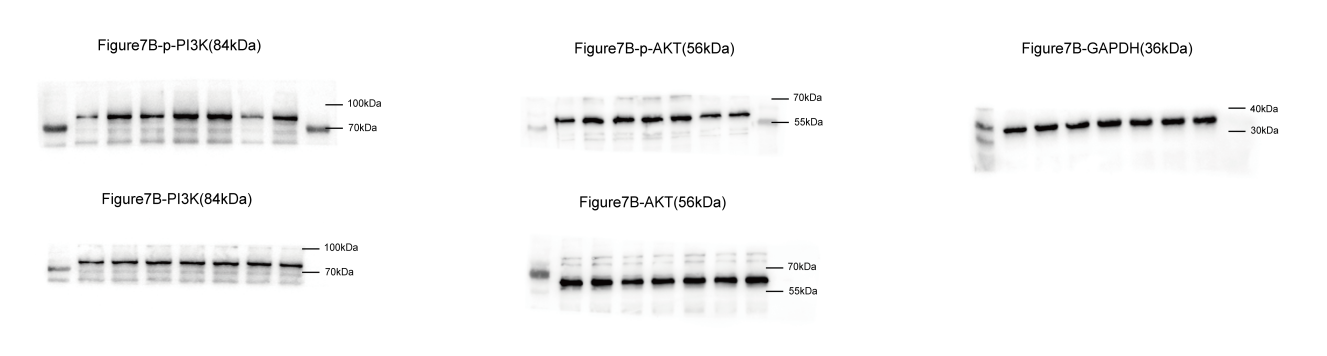


Figure S1


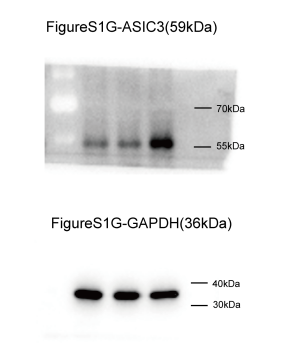


Figure S2


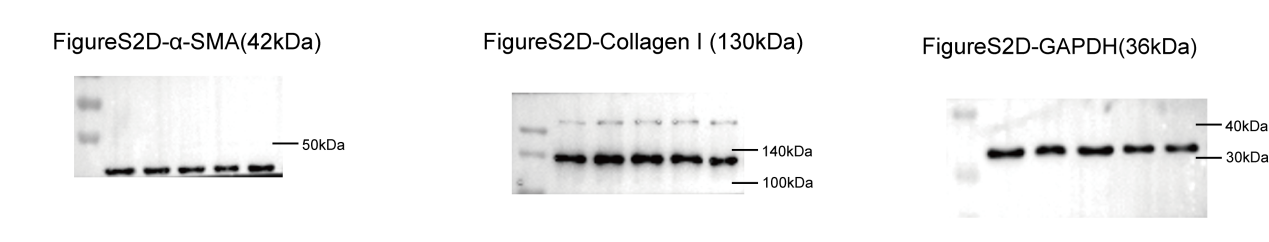

Supplement: Supplementary file 6 — Original Data File [file 41419_2022_4981_MOESM6_ESM.docx]
